# Changing Trends in the Global Consumption of Treatments Used in Hospitalized Patients for COVID-19: A Time Series Multicentre Study

**DOI:** 10.3390/antibiotics12050809

**Published:** 2023-04-25

**Authors:** Judit Aranda, Jose Loureiro-Amigo, Anna Murgadella, Núria Vàzquez, Lucía Feria, Miriam Muñoz, Ariadna Padulles, Gabriela Abelenda, Carol Garcia-Vidal, Montse Tuset, Marta Albanell, Lucía Boix-Palop, Núria Sanmartí-Martínez, Sílvia Gómez-Zorrilla, Daniel Echeverria-Esnal, Alicia Rodriguez-Alarcón, Beatriz Borjabad, Ana Coloma, Jordi Carratalà, Isabel Oriol

**Affiliations:** 1 Infectious Diseases Department, Complex Hospitalari Moisès Broggi, 08970 Sant Joan Despí, Spain; jose.loureiroamigo@sanitatintegral.org (J.L.-A.); nuria.vazquezpiqueras@sanitatintegral.org (N.V.); lucia.feriacasanova@sanitatintegral.org (L.F.); beatriz.borjabadgonzalez@sanitatintegral.org (B.B.); ana.coloma@sanitatintegral.org (A.C.); 2 Pharmacy Department, Complex Hospitalari Moisès Broggi, 08970 Sant Joan Despí, Spain; anna.murgadella@sanitatintegral.org; 3 Pharmacy Department, Hospital Universitari de Bellvitge, 08907 L’Hospitalet de Llobregat, Spain; mmunoz@bellvitgehospital.cat (M.M.); apadulles@bellvitgehospital.cat (A.P.); 4 Bellvitge Biomedical Research Institute (IDIBELL), 08907 L’Hospitalet de Llobregat, Spain; jcarratala@ub.edu; 5 Center for Biomedical Research in Infectious Diseases Network (CIBERINFEC), Instituto de Salud Carlos III, 28029 Madrid, Spain; cgarciav@clinic.cat; 6 Infectious Diseases Department, Hospital Universitari de Bellvitge, 08907 L’Hospitalet de Llobregat, Spain; gabi.abelenda.alonso@gmail.com; 7 Infectious Diseases Department, Hospital Clínic de Barcelona, 08036 Barcelona, Spain; 8 Pharmacy Department, Hospital Clínic de Barcelona, 08036 Barcelona, Spain; mtuset@clinic.cat (M.T.); malbanell@clinic.cat (M.A.); 9 Infectious Diseases Department, Hospital Mútua de Terrassa, 08221 Terrassa, Spain; luciaboix@hotmail.com; 10 Pharmacy Department, Hospital Mútua de Terrassa, 08221 Terrassa, Spain; nsanmarti@mutuaterrassa.cat; 11 Infectious Diseases Department, Hospital del Mar, Infectious Pathology and Antimicrobials Research Group (IPAR), Institut Hospital del Mar d’Investigacions Mèdiques (IMIM) (Center Associated with the Universitat Pompeu Fabra), 08003 Barcelona, Spain; sgomezzorrilla@psmar.cat; 12 Pharmacy Department, Hospital del Mar, Parc De Salut Mar, Infectious Pathology and Antimicrobials Research Group (IPAR), Institut Hospital del Mar d’Investigacions Mèdiques (IMIM) (Center Associated with the Universitat Pompeu Fabra), 08003 Barcelona, Spain; dechevarria@psmar.cat (D.E.-E.); arodriguezalarcon@psmar.cat (A.R.-A.); 13 Clinical Science Department, Faculty of Medicine, University of Barcelona, 08036 Barcelona, Spain

**Keywords:** SARS-CoV-2, treatment trends, antiviral drugs, immunomodulatory drugs, antibiotics, COVID-19 treatments

## Abstract

Aim: To analyze trends in the prescription of COVID-19 treatments for hospitalized patients during the pandemic. Methods: Multicenter, ecological, time-series study of aggregate data for all adult patients with COVID-19 treated in five acute-care hospitals in Barcelona, Spain, between March 2020 and May 2021. Trends in the monthly prevalence of drugs used against COVID-19 were analyzed by the Mantel–Haenszel test. Results: The participating hospitals admitted 22,277 patients with COVID-19 during the study period, reporting an overall mortality of 10.8%. In the first months of the pandemic, lopinavir/ritonavir and hydroxychloroquine were the most frequently used antivirals, but these fell into disuse and were replaced by remdesivir in July 2020. By contrast, the trend in tocilizumab use varied, first peaking in April and May 2020, declining until January 2021, and showing a discrete upward trend thereafter. Regarding corticosteroid use, we observed a notable upward trend in the use of dexamethasone 6 mg per day from July 2020. Finally, there was a high prevalence of antibiotics use, especially azithromycin, in the first three months, but this decreased thereafter. Conclusions: Treatment for patients hospitalized with COVID-19 evolved with the changing scientific evidence during the pandemic. Initially, multiple drugs were empirically used that subsequently could not demonstrate clinical benefit. In future pandemics, stakeholders should strive to promote the early implementation of adaptive randomized clinical trials.

## 1. Introduction

Coronavirus disease 2019 (COVID-19) was first reported in China in late December 2019 and quickly evolved into a pandemic of unforeseeable proportions, progressing to infect more than 546 million people and causing more than 6.3 million deaths worldwide to date [1,2]. The magnitude of its impact on health, wider society, and the economy led the global medical and scientific community to respond proportionally to the challenge. Efforts soon focused on advancing knowledge of this new disease by researching effective vaccines and treatments that could halt its spread [3,4,5].

The early stages of the pandemic were characterized by a lack of scientific evidence and the collapse of the healthcare system, requiring most available physicians to be involved in the care of patients with COVID-19 regardless of their previous training. In this chaotic scenario, health care providers developed local protocols for managing hospitalized patients based on treatment protocols for other respiratory viral infections. However, professional medical societies soon established expert guidelines for the management of COVID-19 based on clinical experience and early research. As the accumulated scientific evidence has changed throughout the pandemic, these guidelines have been modified constantly and quickly [6,7,8].

In December 2022, COVID-19 remained the main target of biomedical research, having led to a never seen before phenomenon of nearly 318,985 entries on MEDLINE since its discovery. Although we now have considerably improved knowledge of the disease due to many high-impact multicenter clinical trials [9,10,11], the first months of the pandemic saw many drugs with potential side effects used off-label that subsequently demonstrated little or no scientific evidence of benefit against COVID-19. To date, however, no studies have evaluated treatment trends against COVID-19 over time. Evaluating these changes could improve our understanding of the mistakes made early in the pandemic and the need to promote the advancement of scientific evidence before acting.

This study aimed to analyze the changes in the epidemiology of different treatments used against COVID-19 during the first 15 months of the pandemic.

## 2. Results

### 2.1. Demographic and Mortality Data

Table 1 provides the demographic and clinical data for the total cohort. The participating centers admitted 22,277 patients due to COVID-19, representing 15% of all admissions during the study period. However, there was marked variability in the percentage of monthly hospital admissions due to COVID-19, which was as high as 51% of admissions in April 2020. Approximately 55% were men and the mean age was 62 years (SD, 2.8). The mean length of hospital stay was 13 days (SD, 1.3) and 15% of patients were admitted to the ICU.

Overall mortality was 10.8%, but this also varied throughout the study period.

**Figure 1:**
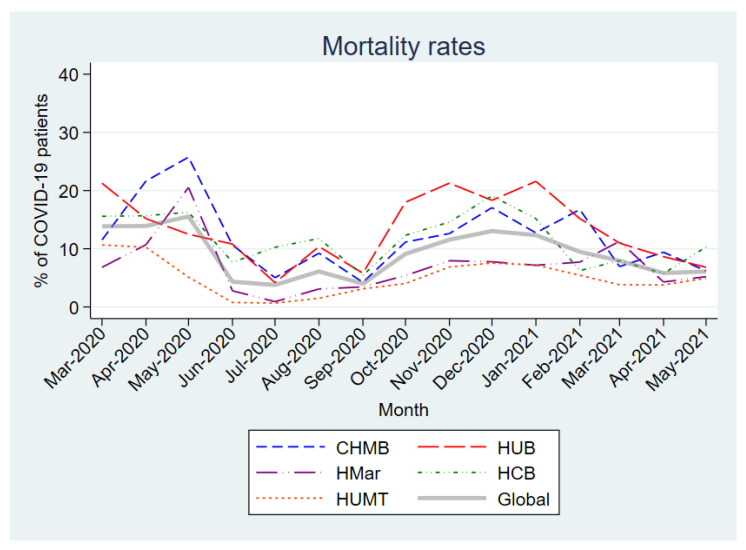
Monthly mortality rates in patients hospitalized with COVID-19. Abbreviations: CHMB: Complex Hospitalari Moisès Borggi; HUB: Hospital Universitari de Bellvitge; HMar: Hospital del Mar; HCB: Hospital Clínic de Barcelona; HUMT: Hospital Universitari Mútua de Terrassa.

Figure 1 shows the overall and center-specific mortality rates for the first 15 months of the pandemic. The first 3 months of the pandemic saw the highest mortality (13.9–15.6%), with further peaks in December 2020 and January 2021 (13.0% and 12.4%, respectively). In addition, many differences in mortality rates were observed between centers.

**Figure 2:**
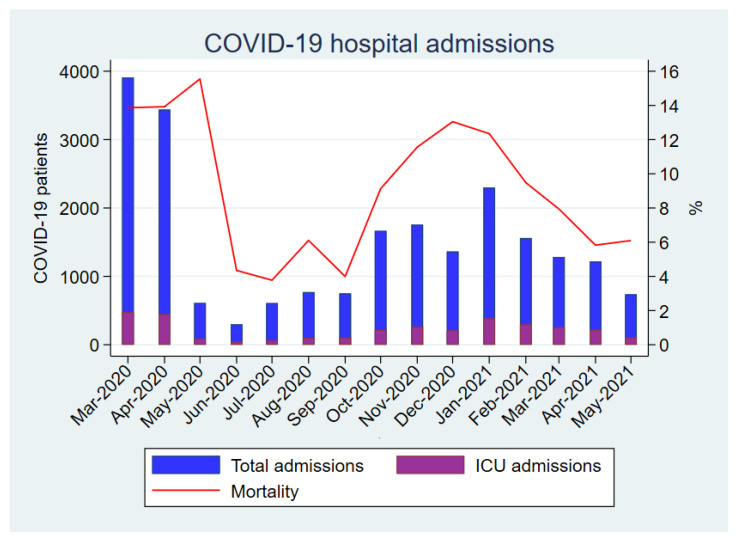
Monthly COVID-19 hospital admissions and global mortality rate in patients hospitalized with COVID-19.

As shown in Figure 2, the crude mortality of patients admitted for COVID-19 was highly variable during the course of the pandemic. The peaks of highest mortality were observed in those periods with the highest incidence of hospital admissions for COVID-19. 

### 2.2. Antiviral Drugs

**Figure 3:**
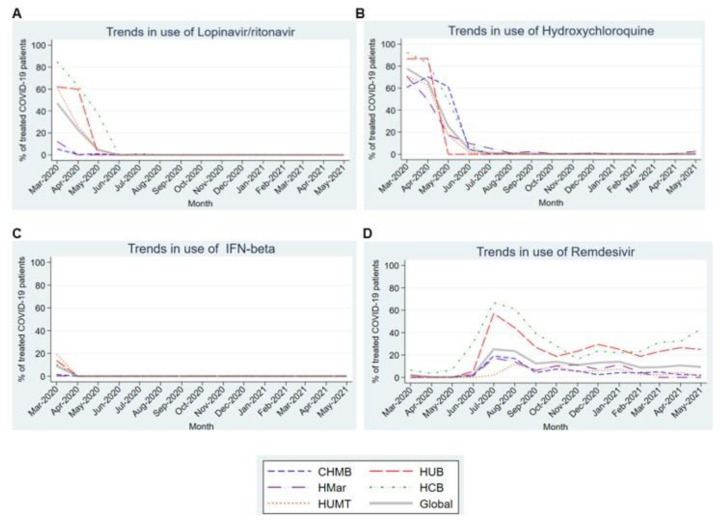
Trends in the use of the different antiviral drugs in the first 15 months of the COVID-19 pandemic. (**A**). Trends in the use of Lopinavir/ritonavir. (**B**). Trends in the use of Hydroxychloroquine. (**C**). Trends in the use of IFN-beta. (**D**). Trends in the use of Remdesivir. Abbreviations: CHMB: Complex Hospitalari Moisès Borggi; HUB: Hospital Universitari de Bellvitge; HMar: Hospital del Mar; HCB: Hospital Clínic de Barcelona; HUMT: Hospital Universitari Mútua de Terrassa.

Figure 3A,B show the high prevalence of lopinavir/ritonavir and hydroxychloroquine use in the first 3–4 months of the pandemic in all participating centers. Note the very marked and statistically significant downward linear trend for both (*p*-values of 0.017 and 0.003, respectively) over the subsequent months until their complete withdrawal. The trend in interferon-beta use was similar (Figure 3C), but with a lower prevalence of use. By contrast, remdesivir (Figure 3D) was scarcely used until July 2020, when it peaked at a prevalence of 25.1% usage before decreasing over the following months. Its use stabilized in the last months of the study, although with differences among the participating centers.

### 2.3. Immunomodulatory and Immunosuppressive Drugs

**Figure 4:**
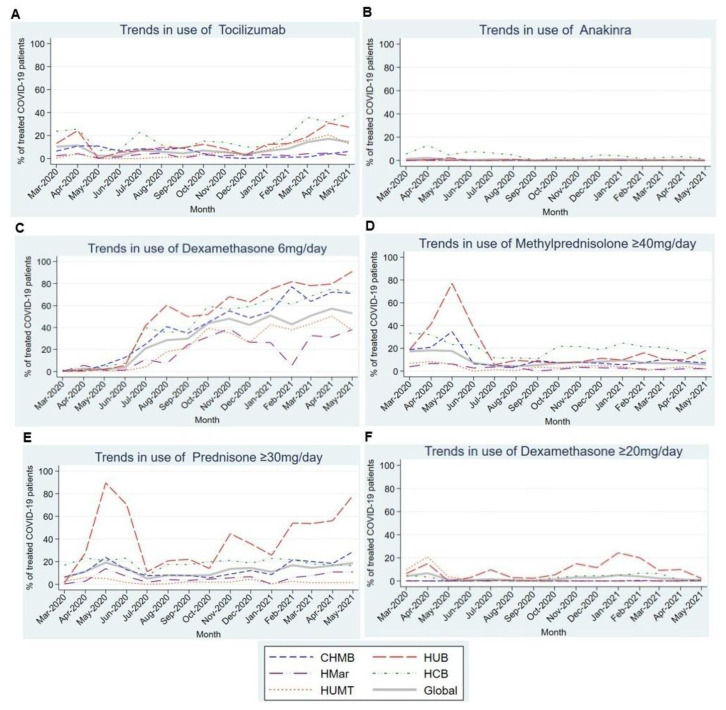
Trends in the use of different immunomodulatory and immunosuppressive drugs in the first 15 months of the COVID-19 pandemic. (**A**). Trends in the use of Tocilizumab in the first 15 months of the COVID-19 pandemic. (**B**). Trends in the use of Anakinra in the first 15 months of the COVID-19 pandemic. (**C**). Trends in the use of Dexamethasone 6mg/day in the first 15 months of the COVID-19 pandemic. (**D**). Trends in the use of Methylprednisolone ≥ 40 mg/day in the first 15 months of the COVID-19 pandemic. (**E**). Trends in the use of Prednisone ≥ 40 mg/day in the first 15 months of the COVID-19 pandemic. (**F**). Trends in the use of Dexamethasone ≥ 20 mg/day in the first 15 months of the COVID-19 pandemic. Abbreviations: CHMB: Complex Hospitalari Moisès Borggi; HUB: Hospital Universitari de Bellvitge; HMar: Hospital del Mar; HCB: Hospital Clínic de Barcelona; HUMT: Hospital Universitari Mútua de Terrassa.

Figure 4A shows the trend in tocilizumab use, highlighting increased use during the first 2 months of the pandemic (10.3% and 11.5%) followed by decreased usage until January 2021. Thereafter, usage began to increase over the following months. Most centers reported near-anecdotal use of anakinra (Figure 4B), except for HCP, where its usage ranged from 4.7% to 13% in the first 5 months of the pandemic.

Regarding corticosteroids, we observed a statistically significant upward linear trend in the use of dexamethasone 6 mg/day (*p* = 0.001) in all participating centers (Figure 4C). By contrast, the use of other corticosteroids showed a stable trend throughout most centers, except in the HUB, where corticosteroid use peaked in the first months of the pandemic (Figure 4D–F).

### 2.4. Antibiotics

**Figure 5:**
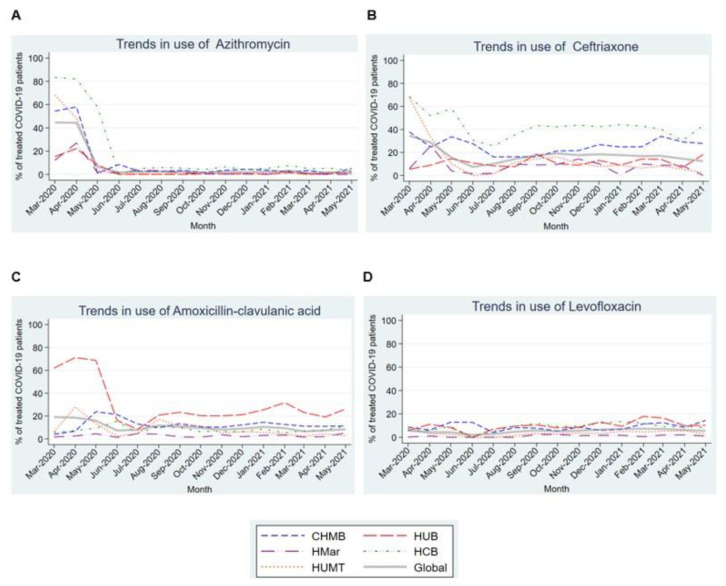
Trends in the use of different antibiotics in the first 15 months of the COVID-19 pandemic. (**A**). Trends in the use of Azythromycin in the first 15 months of the COVID-19 pandemic. (**B**). Trends in the use of Ceftriaxone in the first 15 months of the COVID-19 pandemic. (**C**). Trends in the use of Amoxicillin-clavulanic acid in the first 15 months of the COVID-19 pandemic. (**D**). Trends in the use of Levofloxacin in the first 15 months of the COVID-19 pandemic. Abbreviations: CHMB: Complex Hospitalari Moisès Borggi; HUB: Hospital Universitari de Bellvitge; HMar: Hospital del Mar; HCB: Hospital Clínic de Barcelona; HUMT: Hospital Universitari Mútua de Terrassa.

Figure 5 shows the usage trends for different antibiotics in patients admitted with COVID-19. Nearly 50% of patients in all participating centers received azithromycin in the first three months of the pandemic, before a very marked and statistically significant (*p* = 0.015) downward linear trend over subsequent months to a stable nadir around 1% (Figure 4A). As for the remaining antibiotics, ceftriaxone and amoxicillin-clavulanic also showed maximum use in the first 3 months of the pandemic, although a statistical significance for linear trend was not demonstrated for ceftriaxone. Levofloxacin use ranged between 2% and 7.3% across the analyzed period (Figure 4B–D). 

## 3. Discussion

This time-series analysis described the profound changes in the therapeutic approach for patients with hospitalized COVID-19 during the first 15 months of the pandemic, reflecting the advances in scientific evidence on the disease.

Although the aim of our study was to assess changes over time in trends in the use of COVID-19 treatments, it is worth devoting a paragraph to the evolution of crude hospital mortality during the first 15 months of the pandemic.

In our study, more than 20,000 patients were admitted for COVID-19, representing 15% of all admissions during the study period, and overall mortality was 10.8%, but this varied wildly over the course of the pandemic. 

On the one hand, although there are obviously many factors involved in the incidence of mortality, it can be assumed that a better understanding of the disease, the emergence of effective treatments, the impact of vaccination and the differences between virus strains have played an important role in the decline in the incidence of COVID-19 mortality.

On the other hand, in our study, the highest peaks in mortality were observed in the periods with the highest incidence of hospital admissions for COVID-19. This is probably a consequence of the overload of the healthcare system during the peaks, although, in the case of autumn and winter 2020, an increase in decompensation of comorbidities already commonly seen in cold seasons probably also played a role [12,13].

Finally, to conclude the section related to mortality, different studies have shown that most of the drugs used against COVID-19 showed adverse effects that could also have played a role in the evolution and outcomes of patients admitted for COVID-19 [14,15].

Regarding antiviral treatments, patients mostly received lopinavir/ritonavir and/or hydroxychloroquine during the first months of the pandemic before this practice fell into disuse in July 2020 when remdesivir became the most widely used antiviral treatment. The initial use of lopinavir/ritonavir, and hydroxychloroquine, probably resulted from the known in vitro efficacy against phylogenetically similar viruses such as SARS-CoV and MERS [16,17,18]. Subsequent publications [19,20] demonstrating that these had slight to no impact on mortality, length of hospital stay, or ICU admission among patients hospitalized for COVID-19 and an unfavorable toxicity profile will then have caused their disuse.

Following the publication of the preliminary results of the ACTT-1 Clinical Trials [10], remdesivir was approved by the EMA [21] and its use was recommended in clinical guidelines [6]. However, despite an initial peak in July 2020, usage remained around 10% throughout the study period. We hypothesize that this plateau resulted from most patients being admitted after the initial seven days of symptoms when the drug was indicated during later waves of the pandemic. The lack of clear benefit in patients requiring mechanical ventilation may also have played an important role [10]. Consistent with this, a recent review has been published that evaluated the effects of remdesivir in patients with COVID-19, which concluded that remdesivir probably has little or no effect on all-cause mortality or hospital mortality in individuals with moderate to severe COVID-19 [22]. When discussing the use of Remdesivir, it is important to note that its use in patients with advanced chronic kidney disease was restricted. However, some publications are now appearing that support the use of this drug in this selected group of patients. Probably, if this information had been available in the first 15 months of the pandemic, treatment with remdesivir would have been used in a greater number of cases [23]. 

Finally, although our study focused on hospitalized patients, it is interesting to mention the benefits that treatment with remdesivir has demonstrated in outpatients with COVID-19, in terms of decreased disease progression and poor evolution and decreased need for treatment [24].

The use of tocilizumab in patients with COVID-19 who develop acute respiratory distress syndrome (ARDS) has been controversial. In April and May 2020, the first publications supporting its use in patients with severe COVID-19 appeared [11,21] as an approach to quell the cytokine storm in the hyperinflammatory disease phase [25,26,27]. This corresponded to the first peak in tocilizumab use in our study, with the subsequent decreased use probably related to the publication of contradictory results [28]. A second increase in its use was observed in April and May 2021 after the publication of a meta-analysis by Selvaraj et al. [29] that reported its benefits for mortality, mechanical ventilation requirements, and length of hospital stay in patients with severe COVID-19. In the latest WHO publication in February 2022 [30], tocilizumab was recommended for patients with severe or critical COVID-19. It will be interesting to assess the trend in the use of this drug as the pandemic evolves.

Corticosteroid use in the first months of the pandemic also generated much controversy [31,32,33]. On the one hand, concern existed about their harmful effects based on the previous evidence in respiratory infections like influenza [34]. On the other hand, they had recognized benefit in the management of ARDS [32], and early reports from China suggested that methylprednisolone had a beneficial effect on COVID-19 [33]. Finally, dexamethasone at a dosage of 6 mg per day has been the most widely accepted treatment since July 2020, when the RECOVERY Collaborative Group demonstrated that it decreases the 28-day mortality in patients with COVID-19 requiring oxygen [35].

Antibiotic treatment was widely recommended in COVID-19 management protocols at the beginning of the pandemic. This was due to the parallels drawn between COVID-19 and other respiratory viruses (e.g., H1N1 and H3N2 influenza), which were associated with a high number of bacterial co-infections. However, subsequent studies [36] reported an incidence of less than 10% of bacterial coinfection in patients with COVID-19 during the first months of the pandemic, leading to the conclusion that most of these patients might not require empirical antibacterial treatment. Consistent with this, antibiotic use in our cohort decreased after the first 2 months of the pandemic. Special mention should be made regarding the systematic use of azithromycin in the first wave of the pandemic, with its inclusion in COVID-19 treatment protocols because of its immunomodulatory effect and not for the suspicion of bacterial coinfection. Later research confirmed that this antibiotic offered no benefit for patients with COVID-19 [37], so its use was abandoned.

The strengths of our study are that it was composed of a large cohort of patients hospitalized for COVID-19, the study design was multicenter, and it comprised a long study period. However, it also has important limitations. First, we used a retrospective design and did not collect individual patient data, precluding any assessment of the adequacy of each treatment. Second, because we analyzed aggregated data, we do not know how many patients received more than one antibiotic or corticosteroid. Third, we could not obtain reliable data on the total number of patients hospitalized each month, so we used the monthly number of admissions to calculate the prevalence of use for each drug. Although this should not change the usage trends because we analyzed all drugs in this way, it will probably have overestimated the monthly prevalence.

## 4. Methods

### 4.1. Study Design and Population

We conducted a large, multicenter, ecological time-series study to explore the prevalence of antiviral, immunomodulatory and immunosuppressant, and antibiotic treatment use among patients with COVID-19 hospitalized between 1 March 2020, and 31 May 2021. Data were aggregated for all adult patients with COVID-19 admitted to five acute care hospitals with intensive care units (ICU) in the province of Barcelona, Spain: Complex Hospitalari Moisès Broggi (CHMB), Hospital Universitari de Bellvitge (HUB), Hospital del Mar (HMar), Hospital Clínic de Barcelona (HCB), and Hospital Universitari Mútua de Terrassa (HUMT). Cases of COVID-19 were confirmed by reverse transcription polymerase chain reaction. Patients under 18 years of age and those who were only treated in the emergency department without subsequent admission were excluded from the study.

### 4.2. Data Collection

Researchers collected data directly from the electronic health records of each participating center for patients admitted with COVID-19. This included demographics (age, sex), treatments (lopinavir–ritonavir, hydroxychloroquine, interferon-beta, remdesivir, tocilizumab, anakinra, dexamethasone 6 mg/day, dexamethasone ≥ 20 mg/day, prednisone ≥ 30 mg/day, azithromycin, ceftriaxone, amoxicillin-clavulanic, and levofloxacin), length of hospital stay (LOS), and clinical outcomes, including ICU admission and death during admission. We measured drug consumption based on the monthly prevalence of prescribed treatments (patients who received the drug at some point in the month divided by the number of patients admitted during that month).

Each center followed its own treatment protocols at the start of the pandemic and later adapted them according to the protocols and clinical guidelines that appeared over time. However, the final treatment decision for each patient remained with the treating physician.

### 4.3. Definitions

SARS-CoV-2 infection was confirmed by RT-PCR or SARS-CoV-2 antigen test from a nasopharyngeal swab, sputum, or other respiratory samples. LOS was described as the total number of days from admission to hospital discharge. Mortality was defined as death from any cause during COVID-19 admission.

### 4.4. Statistical Analysis

Continuous variables are presented as means and standard deviations (SD), whereas categorical variables are expressed as numbers and percentages. Monthly incidences rates and their respective 95% confidence intervals were estimated for the following groups: patients with COVID-19 admitted to ICU, patients admitted with COVID-19, patients admitted without COVID-19, and death from COVID-19. We used the non-parametric Cuzick Trend Test to analyze linear trends in the overall monthly prevalence of use of drugs. We used the Mantel–Haenszel test to analyze linear trends in the overall monthly prevalence of use of drugs. All statistical were two-tailed and performed in Stata 15.2 (STATA Corp., College Station, TX, USA) with statistical significance set at *p* < 0.05.

## 5. Conclusions

In summary, the treatment of hospitalized patients with COVID-19 evolved considerably throughout the pandemic, in line with the scientific evidence available at each stage, allowing for the optimization of COVID-19 pharmacological treatment within a few months despite it being a completely new infection. At the beginning of the pandemic, multiple drugs were empirically used that subsequently could not demonstrate clinical benefit. Therefore, while the high mortality in the first part of the pandemic should be considered multifactorial, we suspect the adverse effects of these drugs may have played a role in early outcomes. In future pandemics, stakeholders should strive to promote the early implementation of adaptive randomized clinical trials with the goal of providing clinicians with the best scientific evidence as soon as possible.

## Figures and Tables

**Table 1:** Global demographics by month during the acute COVID-19 episode.

Month	Hospital Occupancy Due to COVID-19 N—% (CI 95%)	COVID-19 Patients Admitted to the ICU % (CI 95%)	Male % (CI 95%)	Age Mean (sd)	COVID-19 Deaths % (CI 95%)	LOS Median of Days (IQR)
March 2020	3909—36.08% (35.18–36.99)	12.23 (11.20–13.26)	54.44 (52.88–56.00)	61.89 (4.08)	13.87 (12.78–14.95)	12.3 (4.79)
April 2020	3440—51.21% (50.01–52.40)	12.99 (11.87–13.26)	54.48 (52.81–56.14)	63.07 (5.08)	13.92 (12.77–15.08)	11.4 (1.23)
May 2020	611—7.98% (7.37–8.58)	14.73 (11.92–17.54)	52.54 (48.58–56.50)	64.18 (6.96)	15.55 (12.67–18.42)	11.5 (5.31)
June 2020	299—3.26% (2.90–3.62)	15.05 (11.00–19.10)	51.17 (45.50–56.84)	61.32 (2.95)	4.35 (2.04–6.66)	11.8 (3.52)
July 2020	609—6.25% (5.77–6.73)	10.51 (8.07–12.94)	54.35 (50.40–58.31)	57.45 (5.34)	3.78 (2.26–5.29)	11.7 (1.14)
August 2020	770—10.60% (9.89–11.30)	13.12 (10.73–15.50)	56.36 (52.86–59.87)	58.70 (4.17)	6.10 (4.41–7.79)	10.6 (3.21)
September 2020	751—8.55% (7.97–9.14)	13.85 (11.38–16.32)	53.93 (50.36–57.49)	59.06 (5.35)	3.99 (2.59–5.40)	13.2 (2.22)
October 2020	1666—14.23% (13.60–14.86)	13.03 (11.41–14.64)	53.66 (51.27–56.06)	61.84 (5.64)	9.12 (7.74–10.51)	12.4 (2.71)
November 2020	1756—15.18% (14.52–15.83)	15.21 (13.53–16.88)	53.82 (51.48–56.06)	64.40 (5.46)	11.56 (10.06–13.06)	12.0 (3.77)
December 2020	1364—13.59% (12.92–14.26)	15.40 (13.48–17.31)	53.30 (50.65–55.95)	66.35 (6.55)	13.05 (11.26–14.84)	14.6 (0.66)
January 2021	2300—22.36% (21.55–23.16)	16.74 (15.21–18.26)	55.39 (53.36–57.42)	65.94 (3.69)	12.35 (11.00–13.69)	12.8 (4.06)
February 2021	1561—15.05% (14.36–15.74)	19.09 (17.14–21.04)	54.64 (52.17–57.11)	64.69 (3.37)	9.48 (8.03–10.93)	12.0 (1.09)
March 2021	1219—10.90% (10.34–11.47)	19.94 (17.75–22.12)	56.00 (53.28–58.71)	62.78 (2.24)	7.94 (6.46–9.42)	11.9 (3.34)
April 2021	9763—11.10% (10.51–11.69)	18.05 (15.89–20.21)	53.40 (50.60–56.20)	61.08 (2.27)	5.82 (4.51–7.14)	13.1 (1.93)
May 2021	10,683—6.46% (6.01–6.91)	14.50 (11.96–17.04)	62.20 (58.70–65.69)	57.96 (0.53)	6.10 (4.37–7.82)	10.1 (1.99)

Abbreviations: ICU: Intensive Care Unit; LOS: length of hospital stay.

## Data Availability

Not applicable.
